# Single-port robotic segmentectomy using the da Vinci SP system for non-small cell lung cancer

**DOI:** 10.3389/fsurg.2026.1811255

**Published:** 2026-07-07

**Authors:** Jun Hee Lee, Hyeong Hun Song, Byung Mo Gu, Soon Young Hwang, Kook Nam Han, Hyun Koo Kim

**Affiliations:** 1 Department of Thoracic and Cardiovascular Surgery, Korea University Guro Hospital, Korea University College of Medicine, Seoul, Republic of Korea; 2 Department of Medicine, Korea University College of Medicine, Seoul, Republic of Korea; 3 Department of Biostatistics, Korea University College of Medicine, Seoul, Republic of Korea; 4 Department of Thoracic and Cardiovascular Surgery, Chung-Ang University Gwangmyeong Hospital, Gwangmyeong, Republic of Korea

**Keywords:** robotics, segmentectomy, single-port, single-port robotic system, uniportal

## Abstract

**Objectives:**

Robot-assisted thoracic surgery using the single-port robotic system is a novel minimally invasive approach; however, clinical data on its use for anatomical segmentectomy remain limited. Therefore, this single-center retrospective cohort study aimed to investigate the safety and feasibility of this technique for segmentectomy by comparing its perioperative outcomes with those of multi-port robot-assisted thoracic surgery and video-assisted thoracoscopic surgery.

**Methods:**

Data from patients with non-small cell lung cancer who underwent anatomical segmentectomy from April 2014 to April 2025 were analyzed. Patients were categorized into single-port and multi-port robot-assisted thoracic surgery groups and video-assisted thoracoscopic surgery group according to the surgical approach. Perioperative outcomes were analyzed after propensity score matching.

**Results:**

A total of 345 patients were included in the analysis: single-port robot-assisted thoracic surgery (SP-RATS, *n* = 50), multi-port robot-assisted thoracic surgery (MP-RATS, *n* = 75), and video-assisted thoracoscopic surgery (VATS, *n* = 220). Following matching, 47 patients were included in each group. All patients in the single-port robot-assisted thoracic surgery group underwent complete resection (R0) without conversion to open thoracotomy. In the matched cohort, the SP-RATS group demonstrated a significantly shorter total operative time than that of the MP-RATS group (*p* < 0.001), whereas no significant difference was observed between the SP-RATS and the VATS groups. In addition, the SP-RATS group had a significantly shorter duration of chest tube drainage than those of the MP-RATS and VATS groups (*p* = 0.015 and *p* = 0.039, respectively). No significant differences were observed between groups in terms of postoperative complications, postoperative pain, or pathological outcomes, including the number of harvested lymph nodes.

**Conclusions:**

Robotic segmentectomy using the da Vinci SP system appears safe and feasible, with favorable short-term perioperative outcomes. Large-scale, well-designed prospective trials with long-term follow-up are remained warranted to validate its clinical efficacy.

## Introduction

Lobectomy has been the standard of care for patients with early-stage non-small cell lung cancer (NSCLC) (1). The increased frequency of low-dose chest computed tomography (CT) screening has increased early identification of small pulmonary nodules or ground-glass opacity lesions (2), leading to a shift toward segmentectomy, although its role as an alternative to lobectomy remains controversial. However, a recent large-scale randomized controlled trial demonstrated that segmentectomy is noninferior to lobectomy in terms of short- and long-term outcomes (3–5). Consequently, the proportion of segmentectomies performed for early-stage NSCLC has continued to increase (6, 7).

In recent years, the application of robotic-assisted thoracic surgery (RATS) for anatomical pulmonary resection has increased (8). Compared to video-assisted thoracoscopic surgery (VATS), RATS can offer several benefits, including three-dimensional visualization, tremor filtration, improved ergonomics, and enhanced instrument articulation. These features enhance the surgical view, enabling surgeons to perform procedures with greater precision and comfort (9). Anatomical segmentectomy is technically more demanding than lobectomy, as it requires meticulous dissection and resection at the segmental level; therefore, RATS may be the optimal option for segmentectomy.

The single-port (SP) robotic surgery platform, based on the da Vinci SP robotic system (Intuitive Surgical, Inc., Sunnyvale, CA, USA), was introduced in 2014 for transoral and urologic procedures. In thoracic surgery, our group was the first to report an initial clinical experience using this system in 2022 (10). Although anatomical pulmonary resections using the SP system have been reported, data on its feasibility for segmentectomy remain limited (11–14), and comparative research on this topic is lacking. Therefore, in this study, we evaluated the safety and feasibility of robotic segmentectomy using the SP system by comparing its perioperative outcomes with those of multi-port (MP) RATS using the Xi system (Intuitive Surgical, Inc.) and VATS.

## Patients and methods

### Ethical statement

The Institutional Review Board of the Korea University Guro Hospital approved this study (approval no. 2025GR0272, approval date: 05/22/2025). The requirement for obtaining informed consent was waived owing to the nature of the retrospective study design, in line with institutional guidelines. All procedures were performed in compliance with the ethical guidelines of the Declaration of Helsinki (2013 revision).

### Patients and selection

We retrospectively collected data from patients who underwent anatomical segmentectomy between April 2014 and April 2025 at Korea University Guro Hospital. The primary endpoint was the occurrence of any postoperative complications. The secondary endpoints were the conversion rate, complete resection (R0) rate, surgical margin distance, and the number of dissected lymph nodes.

**Figure 1:**
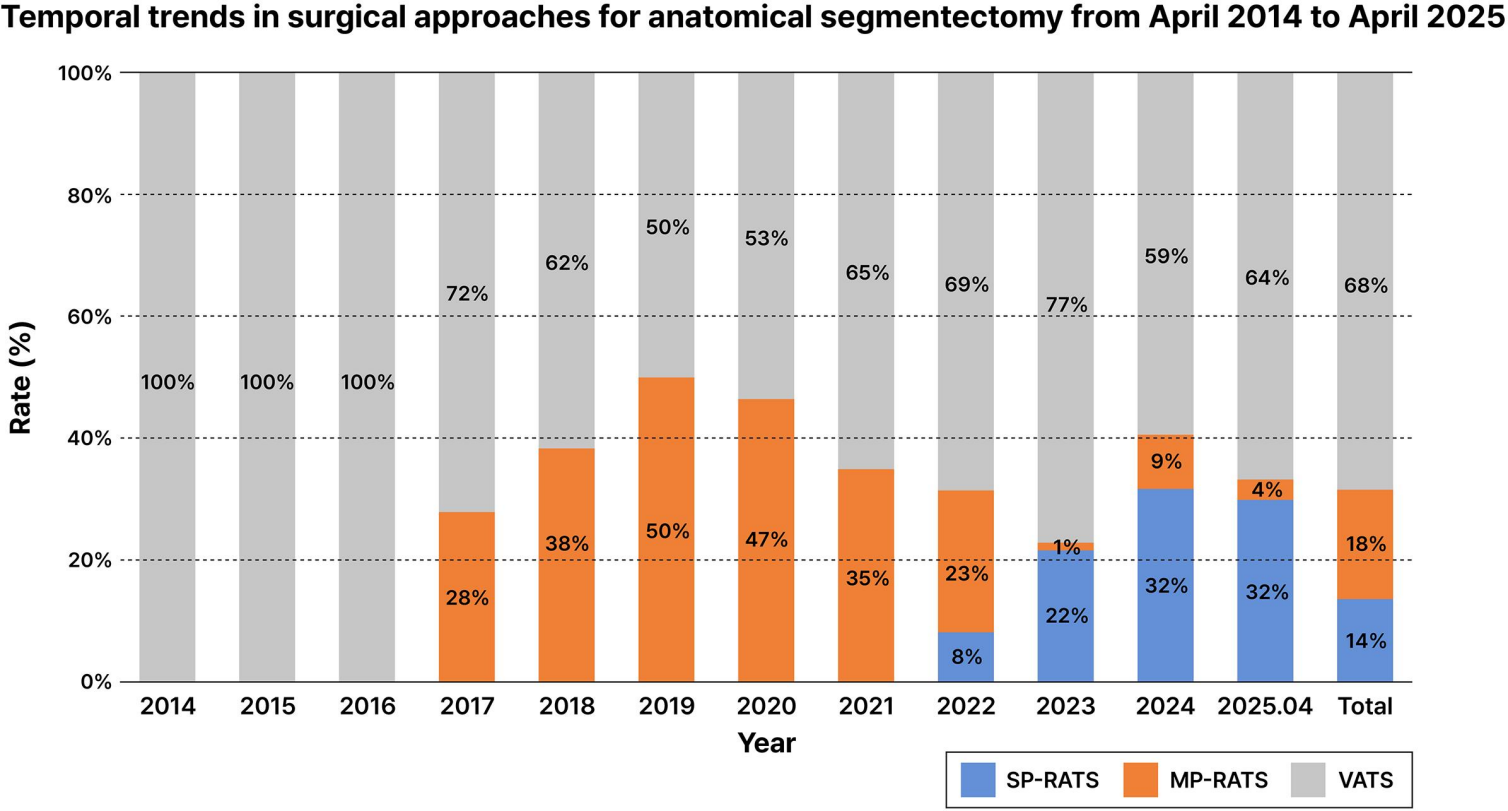
Temporal trends in surgical approaches for anatomical segmentectomy. SP-RATS, single-port robot-assisted thoracic surgery; MP-RATS, multi-port robot-assisted thoracic surgery; VATS, video-assisted thoracic surgery.

Anatomical segmentectomy is defined as the procedure involving resection of ≥1 segment of a pulmonary lobe, achieved through dividing the associated segmental bronchi and arteries in the operative report (15). The decision to proceed with segmentectomy was made through a multidisciplinary review of tumor and clinical characteristics. Based on the surgical approach, the included patients were allocated to the following three groups: SP-RATS, MP-RATS, and VATS. The surgical approach was determined based on patient preference and the surgeon's discretion. In South Korea, the substantial cost difference between VATS and RATS places greater emphasis on patient preference when selecting the surgical approach. Although MP-RATS initially served as the standard robotic approach, SP-RATS has become the predominant approach for robotic segmentectomy since 2023 (Figure 1). Accordingly, the patient's economic status and preference influenced the selection of the surgical approach. Patients who preferred robotic surgery were generally assigned to Surgeon A. All procedures were performed by three thoracic surgeons (H.K.K., J.H.L., and K.N.H.), and all SP-RATS procedures were performed by a single surgeon (H.K.K.) (Supplementary Table S1).

The reason for segmentectomy was categorized as either intentional or compromised. Intentional segmentectomy is typically performed in patients with small (≤2 cm), peripherally located nodules or in those with multiple suspicious pulmonary nodules. Conversely, compromised segmentectomy was indicated for older patients with significant comorbidities, impaired pulmonary or cardiac function, or a history of prior lung surgery (16). The segmentectomy type was classified as either simple or complex: simple segmentectomy involved resection of the left superior segment or right superior segment of the lower lobe, the upper division segment of the left upper lobe, the lingular segment of the left upper lobe, and the entire basal segment of both lower lobes (15–17).

The definition of a postoperative minor complication was based on the Clavien–Dindo classification Grade I–II, whereas a major complication was defined as ≥ Grade IIIa (18). The tumor–node–metastasis (TNM) stage was assessed according to the eighth edition of the American Joint Committee on Cancer TNM classification system.

### Preoperative localization and identification of the segmental plane

Preoperative localization using indocyanine green (ICG) alone, ICG combined with lipiodol, or ICG combined with a microcoil placed under chest CT guidance was performed if the pulmonary nodule was consistent with one or more of the following criteria: (1) nodule size <1 cm; (2) ground-glass or part-solid nodule with a consolidation-to-tumor (C/T) ratio <0.5; (3) deeply located nodule situated >5 mm from the visceral pleura.

The injection site was identified using the fluorescence imaging system and C-arm fluoroscopy. After dividing the targeted bronchus and vessels, 12.5 mg of ICG was injected intravenously, and the intersegmental plane was identified with a fluorescence imaging system (19).

### Operative technique

There were no significant intergroup differences in the extent of systematic mediastinal lymphadenectomy. While mediastinal lymph node dissection was routinely performed, complete lymphadenectomy was occasionally not performed in patients undergoing segmentectomy, especially for compromised indications or in those with pure ground-glass nodules.

### SP-RATS technique

**Figure 2:**
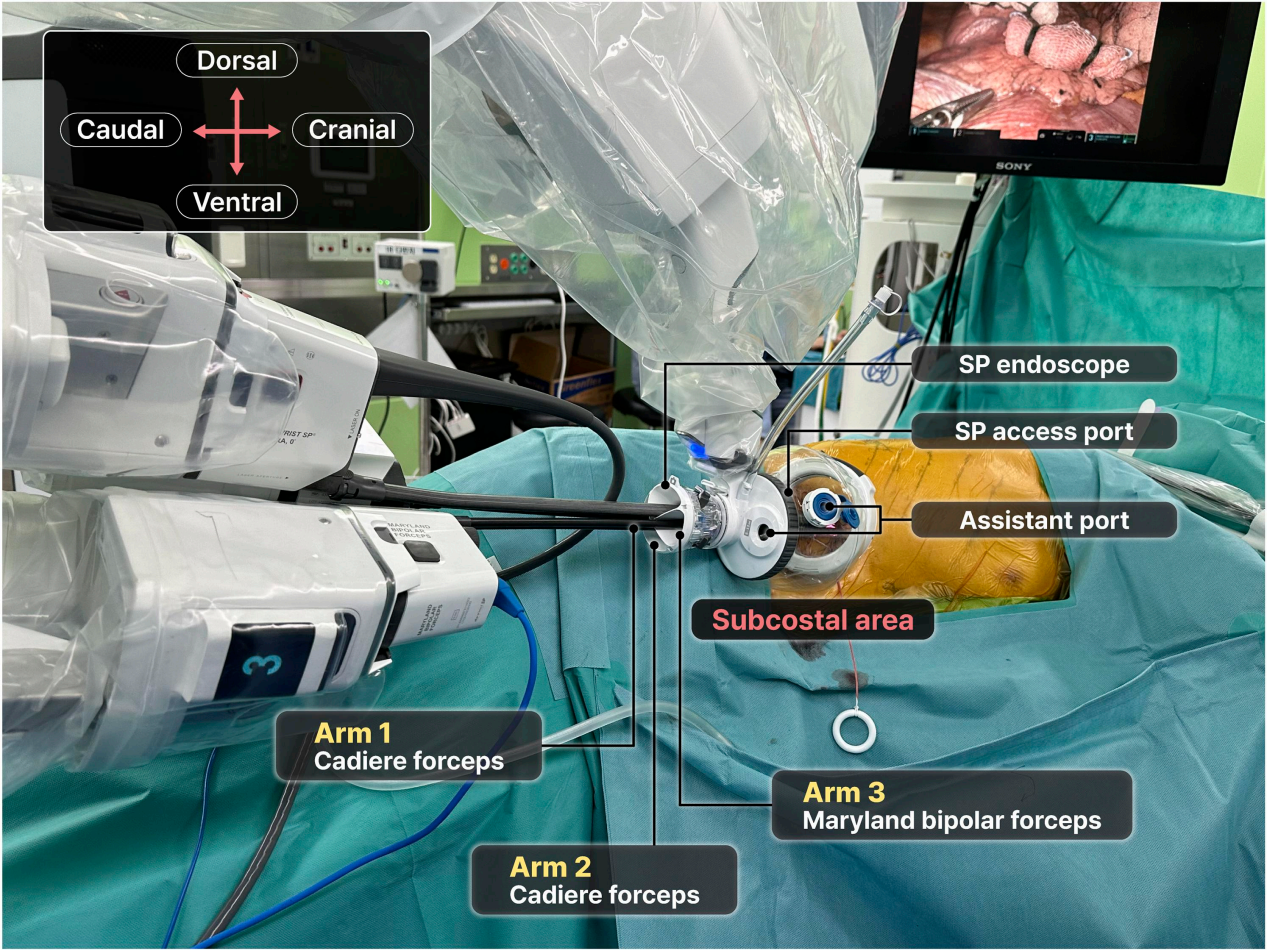
Setting of the da Vinci single-port robotic system for segmentectomy.

The details of the SP-RATS technique using the SP system have been previously demonstrated (12, 20). A single 4 cm incision was placed just anterior to the 6th–8th costal cartilage. After dissecting the two muscle layers and the attachment of the diaphragm, a subcostal tunnel was created, and the SP access port was installed. Unless contraindicated, a pressure of 6–10 mmHg was maintained using carbon dioxide (CO_2_) insufflation. After docking the robotic system, the SP-RATS procedure was performed using three robotic arms (Figure 2). The surgical assistant inserted the instruments to assist with suction, retraction, specimen removal, and stapling. Segmental vessels were preferably divided using a stapler; however, when stapler application was technically challenging, robotic Hem-o-lok clips were favored. If stapler use was deemed difficult after Hem-o-lok clip application, vessel ligation was performed using sutures.

### MP-RATS technique

The standard approach for MP-RATS was a two-port technique (two ports, three arms), consisting of a 4 cm working port at the 7th or 8th intercostal space (ICS) along the posterior axillary line, and a 12 mm port at the 6th or 7th ICS along the anterior axillary line. In the three-port technique (three ports, three arms), an additional 12 mm port was placed at the 8th or 9th ICS along the scapular line. CO_2_ gas was insufflated to maintain a pressure range of 6–10 mmHg.

### VATS technique

SP VATS is our standard approach for VATS segmentectomy. A 2–4 cm incision was created at the 5th ICS to perform the procedure, and an additional port at the 7th ICS was created as necessary.

### Management protocol

There were no differences in chest tube management protocols among the three groups. Chest tube removal was performed once the absence of air leakage was confirmed and when the daily pleural output met our criteria, which was less than 180 mL for patients weighing under 60 kg, or below 3 mL/kg/day for those over 60 kg. If no postoperative complications were observed, patients were typically discharged on the day following chest tube removal.

From the day after surgery, patients were routinely started on oral analgesics, such as acetaminophen with tramadol (two tablets daily) and a nonsteroidal anti-inflammatory agent in an equivalent dosage, unless a contraindication was present. Continuous subpleural infusion of local anesthetics was applied whenever feasible. Postoperative pain intensity was assessed using the Visual Analogue Scale on postoperative days 0, 1, 2, and 3.

### Statistical analysis

Continuous variables were expressed as medians (interquartile ranges) and compared using the independent-samples *t*-test if they followed a normal distribution within each group; otherwise, the Mann–Whitney *U* test was applied for non-normally distributed data within each group. Categorical variables were presented as numbers (percentages), and differences between groups were compared using Pearson's chi-squared test or Fisher's exact test, as appropriate. In addition, we applied the Bonferroni correction to reduce the risk of Type I error. Propensity score matching (PSM) was performed to balance the baseline confounders among the three groups, including age, sex, C/T ratio, indication for segmentectomy, and type of segmentectomy. In the matched dataset, continuous variables were compared using the Kruskal–Wallis test, whereas categorical variables were analyzed using Fisher's exact test. For *post hoc* pairwise comparisons, continuous variables were assessed using the Mann–Whitney *U* test and categorical variables using Fisher's exact test, with the Bonferroni correction applied to account for multiple testing. All statistical analyses were performed using the IBM SPSS software 29.0.2.0 (IBM-SPSS Inc., Armonk, NY, USA). A two-tailed *p*-value <0.05 indicated statistical significance.

## Results

**Figure 3:**
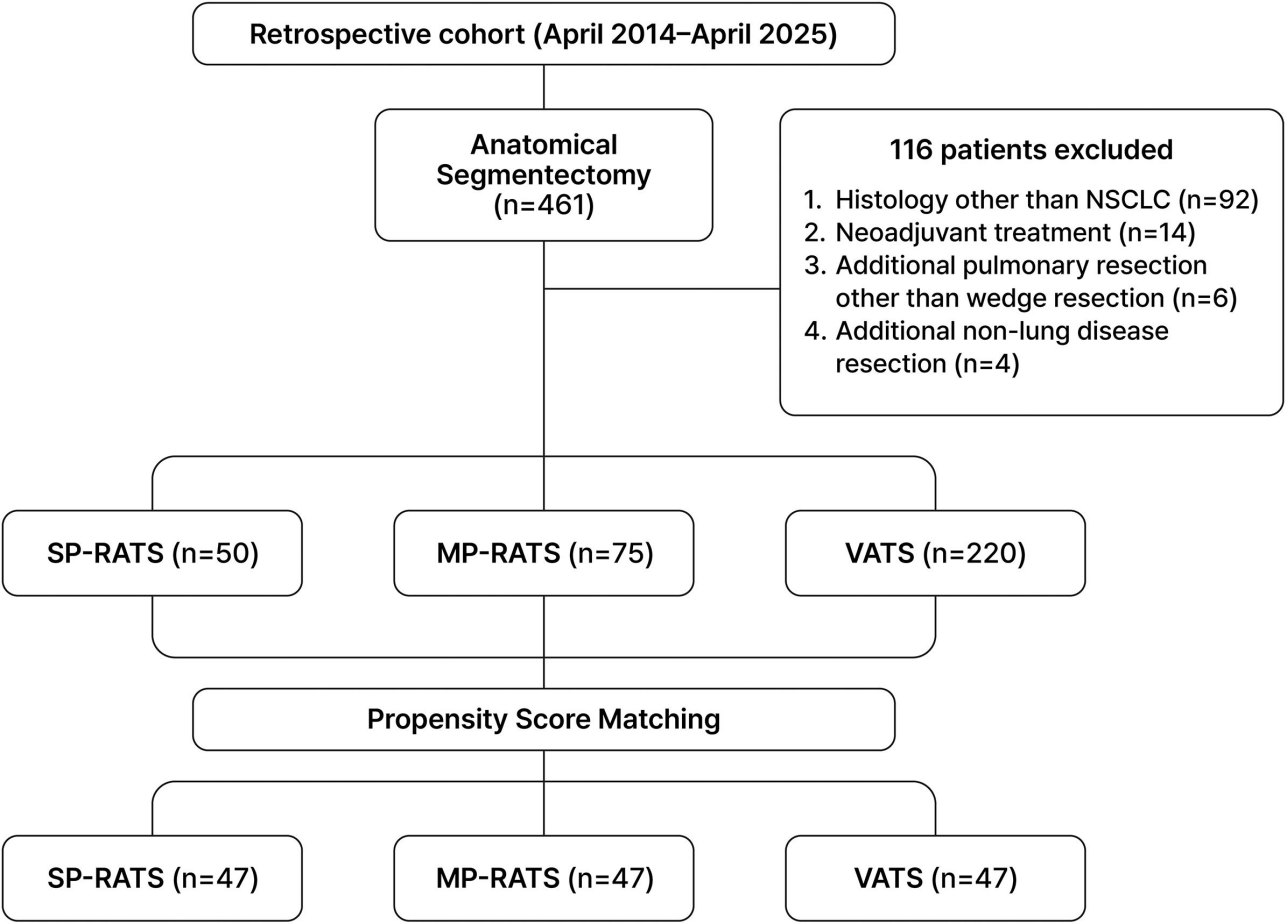
Flow diagram of patient selection. NSCLC, non-small cell lung cancer; SP-RATS, single-port robot-assisted thoracic surgery; MP-RATS, multi-port robot-assisted thoracic surgery; VATS, video-assisted thoracic surgery.

A total of 345 patients were included in the analysis and categorized into three groups: SP-RATS (*n* = 50), MP-RATS (*n* = 75), and VATS (*n* = 220). After PSM, 47 patients were included in each group (Figure 3).

Table 1 lists the baseline characteristics of the patients in each group. Patients who underwent VATS segmentectomy were significantly older than those in the SP-RATS and MP-RATS groups (*p* = 0.003 and *p* < 0.001, respectively). The SP-RATS group demonstrated a higher proportion of patients with a C/T ratio of ≤0.5 than the VATS group (68% vs. 42%, *p* = 0.006). Supplementary Table S2 presents details of the types of segmentectomy. Table 2 presents the baseline characteristics of the matched populations. After PSM, no significant differences in baseline characteristics were observed among the groups.

**Table 1:** Baseline characteristics.

Variables	SP-RATS	MP-RATS	VATS	*p*-value		
	(*n* = 50)	(*n* = 75)	(*n* = 220)	SP vs. MP RATS	SP-RATS vs. VATS	MP-RATS vs. VATS
Age (years)	63 (58–66)	63 (56–70)	68 (61.0–73.7)	1.000	0.003	<0.001
Sex, male	20 (40%)	45 (60%)	122 (55%)	0.084	0.144	1.000
BMI (kg/m^2^)	22.9 (20.6–25.0)	23.7 (21.7–26.3)	23.8 (21.9–26.0)	0.282	0.177	1.000
FEV1 (%)	91 (79.7–98.0)	90 (80.0–99.0)	86 (76.0–97.0)	1.000	0.513	0.372
Tumor size (cm)	1.7 (1.4–2.2)	1.5 (1.2–2.1)	1.5 (1.2–2.1)	1.000	0.720	1.000
C/T ratio				0.306	0.006	0.288
≤0.5	34 (68%)	40 (53%)	93 (42%)			
>0.5	16 (32%)	35 (47%)	127 (58%)			
Tumor location				1.000	1.000	0.804
RUL	18 (36%)	19 (25%)	73 (33%)			
RML	0	0	1 (1%)			
RLL	9 (18%)	14 (19%)	51 (23%)			
LUL	14 (28%)	30 (40%)	59 (27%)			
LLL	9 (18%)	12 (16%)	36 (16%)			
Reason for segmentectomy				1.000	0.786	1.000
Intentional	14 (28%)	23 (31%)	80 (36%)			
Compromise	36 (72%)	52 (69%)	140 (64%)			
Type of segmentectomy				0.426	1.000	1.000
Simple	13 (26%)	29 (39%)	72 (33%)			
Complex	37 (74%)	46 (61%)	148 (67%)			

Data are presented as median (interquartile range) or *n* (%). BMI, body mass index; C/T ratio, consolidation to tumor ratio; FEV1, forced expiratory volume in 1 s; MP, multi-port; RATS, robot-assisted thoracic surgery; SP, single-port; VATS, video-assisted thoracic surgery; RUL, right upper lobe; RML, right middle lobe; RLL, right lower lobe; LUL, left upper lobe; LLL, left lower lobe.

**Table 2:** Baseline characteristics of matched populations.

Variables	SP-RATS	MP-RATS	VATS	*p*-value		
	(*n* = 47)	(*n* = 47)	(*n* = 47)	SP vs. MP RATS	SP-RATS vs. VATS	MP-RATS vs. VATS
Age (years)	64 (59–67)	63 (56–72)	64 (59–71)	1.000	1.000	1.000
Sex, male	20 (43%)	23 (49%)	22 (47%)	1.000	1.000	1.000
BMI (kg/m^2^)	22.9 (20.6–25.1)	23.2 (21.4–26.8)	24.0 (22.0–26.6)	1.000	0.107	0.789
FEV1 (%)	91 (80.0–98.0)	90 (80.0–98.0)	89 (78.0–98.0)	1.000	1.000	1.000
Tumor size (cm)	1.7 (1.3–2.2)	1.6 (1.3–2.1)	1.6 (1.2–2.0)	1.000	1.000	1.000
C/T ratio				1.000	1.000	1.000
≤0.5	31 (66%)	33 (70%)	34 (72%)			
>0.5	16 (34%)	14 (30%)	13 (28%)			
Tumor location				1.000	1.000	1.000
RUL	17 (36%)	14 (30%)	16 (34%)			
RLL	9 (19%)	8 (17%)	8 (17%)			
LUL	12 (26%)	17 (36%)	14 (31%)			
LLL	9 (19%)	8 (17%)	9 (18%)			
Reason for segmentectomy				1.000	1.000	1.000
Intentional	14 (30%)	13 (28%)	14 (30%)			
Compromise	33 (70%)	34 (72%)	33 (70%)			
Type of segmentectomy				1.000	1.000	1.000
Simple	14 (30%)	13 (28%)	14 (30%)			
Complex	33 (70%)	34 (72%)	33 (70%)			

Data are presented as the median (interquartile range) or *n* (%). BMI, body mass index; C/T ratio, consolidation-to-tumor ratio; FEV1, forced expiratory volume in 1 s; MP, multi-port; RATS, robot-assisted thoracic surgery; SP, single-port; VATS, video-assisted thoracic surgery; RUL, right upper lobe; RML, right middle lobe; RLL, right lower lobe; LUL, left upper lobe; LLL, left lower lobe.

Perioperative outcomes are summarized in Table 3. All patients in the SP-RATS group achieved R0 resection. The total operative time was significantly shorter in the SP-RATS group than in the MP-RATS group (138 [116.5–167.5] vs. 166 [140.0–195.0] min, *p* < 0.001), but did not differ significantly from that of the VATS group. The SP-RATS group also demonstrated a significantly shorter duration of chest tube drainage than the MP-RATS and VATS groups (*p* = 0.024 and 0.015, respectively). Table 4 summarizes the perioperative outcomes of the matched cohorts after PSM. Even after PSM, significant differences in the total operative time and chest tube duration persisted among the groups. The total operative time was significantly shorter in the SP-RATS group than in the MP-RATS group (135 [112.0–167.0] vs. 168 [140.0–195.0] min, *p* = 0.001), but did not differ significantly from that of the VATS group (125 [78–175.0] min, *p* = 0.737). The chest tube duration was significantly shorter in the SP-RATS group than in both the MP-RATS and VATS groups (2 [1–3] vs. 3 [2–5] days, *p* = 0.015; vs. 2 [2–4] days, *p* = 0.039, respectively).

**Table 3:** Perioperative outcomes.

Variables	SP-RATS	MP-RATS	VATS	*p*-value		
	(*n* = 50)	(*n* = 75)	(*n* = 220)	SP vs.MP RATS	SP-RATS vs. VATS	MP-RATS vs. VATS
Resection status				1.000	1.000	1.000
R0	50 (100%)	74 (99%)	215 (98%)			
R 1/2	0	1 (1%)	5 (2%)			
Total operative time (min)	138 (116.5–167.5)	166 (140.0–195.0)	129 (90–162.2)	<0.001	0.429	<0.001
Conversion						
to VATS	1 (2%)	0	N/A	1.000	N/A	N/A
to open	0	0	2 (1%)	N/A	1.000	1.000
Chest tube duration (days)	2 (1–3)	2 (2–4)	2 (2–4)	0.024	0.015	1.000
Postoperative hospital stay (days)	4 (3–6)	4 (3–6)	4 (3–6)	0.870	0.789	1.000
Postoperative pain (VAS)						
POD 0	3 (2–4)	3 (3–5)	3 (3–5)	0.069	0.633	0.153
POD 1	3 (2–3)	3 (2–3)	3 (2–3)	1.000	1.000	1.000
POD 2	2 (1.7–3.0)	3 (2–3)	3 (2–3)	0.195	0.714	0.780
POD 3	2 (1–3)	3 (2–3)	2 (2–3)	0.321	1.000	0.639
Complications				0.963	1.000	1.000
None	44 (88%)	63 (84%)	190 (86%)			
Minor (Grade I–II)	3 (6%)	10 (13%)	22 (10%)			
Major (≥Grade IIIa)	3 (6%)	2 (3%)	8 (4%)			
Prolonged air leakage (days >5)	2 (4%)	5 (7%)	11 (5%)	1.000	1.000	1.000

Data are presented as median (interquartile range) or *n* (%). MP, multi-port; POD, postoperative day; RATS, robot-assisted thoracic surgery; R0, complete resection; R (un), uncertain resection; R1, microscopically positive resection margin; R2, gross unresected tumor remaining; SP, single-port; VAS, visual analogue scale; VATS, video-assisted thoracoscopic surgery.

**Table 4:** Perioperative outcomes of matched populations.

Variables	SP-RATS	MP-RATS	VATS	*p*-value		
	(*n* = 47)	(*n* = 47)	(*n* = 47)	SP vs.MP RATS	SP-RATS vs. VATS	MP-RATS vs. VATS
Resection status				1.000	1.000	1.000
R0	47 (100%)	47 (100%)	46 (99%)			
R1/2	0	0	1 (2%)			
Total operative time (min)	135 (112.0–167.0)	168 (140.0–195.0)	125 (78–175.0)	0.001	0.737	0.003
Conversion						
to VATS	1 (2%)	0	N/A	1.000	N/A	N/A
to open	0	0	1 (2%)	N/A	1.000	1.000
Chest tube duration (days)	2 (1–3)	3 (2–5)	2 (2–4)	0.015	0.039	1.000
Postoperative hospital stay (days)	4 (3–6)	3 (2–5)	2 (2–4)	0.859	1.000	1.000
Postoperative pain (VAS)						
POD 0	3 (2–4)	3 (3–5)	3 (2–5)	0.125	1.000	1.000
POD 1	3 (2–3)	3 (2–4)	3 (2–5)	1.000	0.521	1.000
POD 2	2 (1.7–3.0)	3 (2–3)	3 (2–3)	0.585	0.361	1.000
POD 3	2 (1–3)	3 (2–3)	2 (2–3)	0.318	0.903	1.000
Complications				0.704	0.977	0.694
None	41 (87%)	38 (81%)	43 (92%)			
Minor (Grade I–II)	3 (6%)	8 (17%)	4 (8%)			
Major (≥Grade IIIa)	3 (6%)	1 (2%)	0			
Prolonged air leakage (days >5)	2 (4%)	3 (6%)	2 (4%)	1.000	1.000	1.000

Data are presented as the median (interquartile range) or *n* (%). MP, multi-port; POD, postoperative day; RATS, robot-assisted thoracic surgery; R0, complete resection; R (un), uncertain resection; R1, microscopically positive resection margin; R2, gross remaining unresected tumor; SP, single-port; VAS, visual analogue scale; VATS, video-assisted thoracoscopic surgery.

Table 5 presents the pathological outcomes of each approach. The SP-RATS and MP-RATS groups retrieved a greater number of N1 lymph nodes (LNs) than the VATS group (*p* = 0.042 and *p* < 0.001, respectively), suggesting a more thorough hilar LN evaluation in robotic procedures. The pathological outcomes of the matched cohorts are summarized in Table 6. After PSM, the pathological variables, including the number of dissected lymph nodes, were comparable among the groups.

**Table 5:** Pathological outcomes.

Variables	SP-RATS	MP-RATS	VATS	*p*-value		
	(*n* = 50)	(*n* = 75)	(*n* = 220)	SP vs. MP RATS	SP-RATS vs. VATS	MP-RATS vs. VATS
Surgical margin distance (cm)	1.9 (1.3–2.7)	2.2 (1.4–3.5)	1.9 (1.2–2.7)	0.465	1.000	0.084
Pathological type				1.000	1.000	0.345
ADC	46 (92%)	69 (92%)	189 (86%)			
SCC	4 (8%)	4 (5%)	28 (13%)			
Other	0	2 (3%)	3 (1%)			
cTNM stage				1.000	1.000	1.000
IA	44 (88%)	67 (89%)	187 (85%)			
IB	4 (8%)	7 (9%)	26 (12%)			
II or more	2 (4%)	1 (1%)	7 (3%)			
pTNM stage				0.549	1.000	1.000
0	4 (8%)	1 (1%)	10 (4%)			
IA	40 (80%)	60 (80%)	171 (78%)			
IB	3 (6%)	10 (13%)	26 (12%)			
II or more	3 (6%)	4 (5%)	13 (6%)			
Number of dissected LNs						
Total LNs	9 (4–14)	9 (6–14)	7 (3.0–11.7)	1.000	0.291	0.012
N1 LNs	3.5 (0.7–6.2)	4 (2–5)	2 (0–4)	1.000	0.042	<0.001
N2 LNs	5 (2.0–8.2)	5 (3–8)	4 (1–8)	1.000	1.000	0.327
Nodal upstaging	3 (6%)	0	4 (2%)	0.186	0.363	1.000

Data are presented as median (interquartile range) or *n* (%). ADC, adenocarcinoma; LNs, lymph nodes; MP, multi-port; RATS, robot-assisted thoracic surgery; SCC, squamous cell carcinoma; SP, single-port; VATS, video-assisted thoracoscopic surgery; cTNM, clinical tumor–node–metastasis stage; pTNM, pathological tumor–node–metastasis stage.

**Table 6:** Pathological outcomes of matched populations.

Variables	SP-RATS	MP-RATS	VATS	*p*-value		
	(*n* = 47)	(*n* = 47)	(*n* = 47)	SP vs. MP RATS	SP-RATS vs. VATS	MP-RATS vs. VATS
Surgical margin distance (cm)	1.9 (1.3–2.8)	2.0 (1.4–2.9)	1.9 (1.3–2.9)	1.000	1.000	1.000
Pathological type				1.000	1.000	1.000
ADC	43 (92%)	46 (98%)	44 (94%)			
SCC	4 (8%)	1 (2%)	2 (4%)			
Other	0	0 (0%)	1 (2%)			
cTNM stage				1.000	1.000	1.000
IA	41 (87%)	43 (91%)	43 (91%)			
IB	4 (8%)	4 (8%)	4 (8%)			
II or more	2 (4%)	0	0			
pTNM stage				1.000	1.000	1.000
0	4 (8%)	1 (2%)	3 (6%)			
IA	37 (79%)	37 (79%)	39 (83%)			
IB	3 (6%)	6 (13%)	3 (6%)			
II or more	3 (6%)	3 (6%)	2 (4%)			
Number of dissected LNs						
Total LNs	9 (4–14)	9 (6–15)	7 (3.0–11.0)	1.000	0.707	0.334
N1 LNs	3 (1–6)	4 (2–5)	2 (0–6)	1.000	0.858	0.110
N2 LNs	5 (2–9)	5 (2–8)	4 (2–7)	1.000	1.000	0.943
Nodal upstaging	3 (6%)	0	0	0.725	0.725	1.000

Data are presented as the median (interquartile range) or *n* (%). ADC, adenocarcinoma; LNs, lymph node; MP, multi-port; RATS, robot-assisted thoracic surgery; SCC, squamous cell carcinoma; SP, single-port; VATS, video-assisted thoracoscopic surgery; cTNM, clinical tumor–node–metastasis stage; pTNM, pathological tumor–node–metastasis stage.

Supplementary Table S3 presents the subgroup analysis of patients who underwent complex segmentectomy. In this subgroup analysis, the total operative time remained significantly shorter in the SP-RATS group than in the MP-RATS group (135 [112–162] vs. 164 [129–182] min, *p* = 0.030). The SP-RATS group also demonstrated a significantly shorter duration of chest tube drainage compared with both the MP-RATS and VATS groups (*p* = 0.006 and *p* = 0.045, respectively).

## Discussion

To the best of our knowledge, this study is the first to report an early feasibility experience of SP-RATS segmentectomy using the SP system via the subcostal approach in patients with NSCLC. The SP-RATS group showed comparable perioperative outcomes with favorable results in postoperative pain, total operative time, and duration of chest tube drainage. Therefore, this technique is potentially feasible and a less invasive alternative to MP-RATS and VATS in selected patients.

As a feasible and safe procedure, RATS segmentectomy is gaining traction as a preferred approach for pulmonary segmentectomy. However, controversy remains regarding whether RATS segmentectomy offers superior outcomes to VATS. Using data from the National Cancer Database across the United States, Kodia et al. reported that RATS segmentectomy, compared to open thoracotomy and VATS, was associated with harvesting more LNs and shorter hospital stay (21). Similarly, a multi-institutional study showed increased N1 node and station yields in the RATS group (22).

A key factor for quality assessment in segmentectomy is the number of LNs harvested. Logan et al. included sampling ≥10 LNs as one of the quality indicators for segmentectomy (23). However, in this study, most segmentectomies were performed for compromised reasons, and in such patients, extensive lymph node dissection is often not feasible. Thus, the median number of LNs dissected did not exceed 10 in all groups. Nonetheless, the SP-RATS and MP-RATS approaches retrieved more LNs than VATS before matching, consistent with recent findings (21, 22, 24). Wilson et al. reported that the nodal upstaging rate was higher in the robotic approach than VATS, which likely reflects the robot's ability to facilitate direct dissection of the interlobar fissure and precise removal of hilar LNs along the pulmonary vessels and bronchus, particularly at LN station 11 (25). In addition, the robotic platform provides stable retraction and enhanced three-dimensional visualization, thereby facilitating meticulous and oncologically sound lymph node dissection. This allows for more controlled and complete lymph node dissection, particularly around the interlobar fissure and the LN station 11 (24). Furthermore, this system enables enhanced precision in dissecting and dividing segmental bronchi and vessels, facilitating safer and higher-quality anatomical segmentectomies. Although a higher number of N1 lymph nodes was observed in the robotic groups, the overall lymph node yield remained relatively low, and no statistically significant difference was observed after PSM. This difference may reflect technical factors rather than a true oncologic advantage. Therefore, these results should not be overinterpreted, and further studies are required to determine their clinical significance.

Our SP-RATS approach using the SP robotic system is characterized by two key features: the use of three robotic arms through a single incision, and the adoption of a subcostal approach. In this study, SP-RATS was associated with a significantly shorter total operative time compared to that of the MP-RATS. The MP-RATS procedures were performed earlier in the study period using two robotic arms, whereas the SP-RATS was performed more recently using three robotic arms. Therefore, the observed difference in operative time may be attributable to these technical differences, as well as temporal factors, including the accumulation of surgeon experience. Furthermore, at our institution, the MP-RATS is performed using a two-arm technique rather than the more widely adopted multiport technique, whereas the SP-RATS utilizes a single-port three-arm approach. These non-standard approaches may limit the generalizability of our findings and compromise the validity of direct comparisons between the groups. Accordingly, the shorter operative time observed in the SP-RATS group should be interpreted with caution.

Additionally, in this study, patients in the SP-RATS group experienced a significantly shorter duration of chest tube drainage than those in the MP-RATS and VATS groups. This finding is consistent with that of prior studies reporting shorter chest tube durations in the RATS group than the VATS (24, 26). This result may be attributed to the robotic system's capacity for more precise and delicate surgical maneuvers, which reduce intraoperative bleeding and trauma to surrounding tissues, ultimately decreasing postoperative drainage (24). Furthermore, segmentectomy inherently involves complex intersegmental plane divisions, and less precise dissection may increase the risk of parenchymal injury and subsequent air leakage. The difference in chest tube duration between the SP-RATS and MP-RATS groups may be explained by the surgeon's accumulated experience in robotic surgery and the inherent benefits of the single-port approach, both of which likely contributed to a faster postoperative recovery. Similar trends were consistently observed in the subgroup analysis of complex segmentectomy. In this subgroup, SP-RATS demonstrated significantly shorter total operative time and chest tube duration compared with MP-RATS and VATS, suggesting that the technical advantages of SP-RATS remain effective even in technically demanding segmentectomies.

Postoperative pain is a key factor that facilitates early recovery and significantly impacts a patient's quality of life. The subcostal SP approach may minimize intercostal nerve injury and thereby reduce postoperative pain (27); however, this benefit has not yet been conclusively established. In the present study, no statistically significant difference in postoperative pain was observed among the three groups. These findings should be interpreted with caution, as the assessment of postoperative pain was limited by the relatively small sample size and inherent constraints of the retrospective study design, which may have hindered the precise comparison of pain outcomes. In addition, several important factors that may influence postoperative pain have not been adequately evaluated, including incision size, analgesic protocols, variations in pain management strategies, use of continuous subpleural infusion of local anesthetics, total amount of analgesics administered, and post-discharge pain. Therefore, the current results are insufficient to draw definitive conclusions regarding the effects of surgical approach on postoperative pain. Therefore, large-scale prospective studies are essential to comprehensively evaluate these factors and validate the clinical benefits of the subcostal SP approach.

In this study, the da Vinci SP system was utilized to perform SP-RATS segmentectomy, offering potential benefits, including faster recovery, reduced postoperative pain, and improved cosmetic results. However, this system currently presents some technical challenges. First, the da Vinci SP system lacks a dedicated energy-sealing device. This may prolong operative time and complicate small-vessel division. Additionally, due to the cannula diameter (2.5 cm), a subcostal approach was used in all SP-RATS cases. The most critical technical limitation was that a dedicated robotic stapler for the SP system had not yet been approved in Korea, resulting in stapling being performed by the surgical assistant. Various types of segmentectomy using the SP system were successfully performed without conversion to thoracotomy. However, in certain segments, technical challenges were encountered during stapling of the intersegmental plane. In the subgroup analysis of complex segmentectomy, no statistically significant differences in resection margin distance were observed among the three groups. These findings support the technical feasibility of SP-RATS segmentectomy; however, larger multi-center studies are needed to confirm the reproducibility and generalizability of these results. As technology continues to advance and dedicated robotic staplers gain regulatory approval, SP-RATS segmentectomy may become more widely adopted in clinical practice.

This study has some limitations. The generalizability of the findings is limited because of the single-center, retrospective cohort design and the relatively small sample size. Further larger multi-center studies are needed. Additionally, given its retrospective design over a decade, selection bias and confounding variables may have influenced the results. A time-based subgroup analysis was not feasible because of the substantial temporal imbalance between the groups, with SP-RATS cases concentrated in the later period and MP-RATS and VATS cases predominantly performed in the earlier period. Additionally, both robotic approaches were mainly performed by a single surgeon, potentially introducing surgeon-related bias that may have influenced the perioperative outcomes. As robotic segmentectomy using the SP system has only recently been introduced, this study did not evaluate long-term oncologic outcomes, such as recurrence rates and overall survival, which limits the ability to draw definitive conclusions regarding the overall effectiveness of the technique. Finally, the MP-RATS technique used in this study does not represent the standard MP robotic approach. This may thus limit the generalizability of our findings and complicate direct comparisons between the two techniques.

## Conclusion

Robotic segmentectomy using the SP system appears to be a feasible and safe approach for the treatment of NSCLC with favorable short-term perioperative outcomes. However, the clinical significance of these findings is limited by the lack of long-term oncological outcomes, including recurrence and survival. Therefore, large-scale prospective studies on long-term outcomes are warranted to validate its efficacy.

## Data Availability

The original contributions presented in the study are included in the article/Supplementary Material, further inquiries can be directed to the corresponding author.
