# Supplementary material for: Single-port robotic segmentectomy using the da Vinci SP system for non-small cell lung cancer
Source: Front Surg. 2026 Jul 7;13:1811255. doi: 10.3389/fsurg.2026.1811255 (PMC13386685; doi:10.3389/fsurg.2026.1811255)
Supplement: Supplementary file 1 [file Supplementaryfile1.docx]

Supplementary Table S1. Distribution of surgical approaches for segmentectomy by surgeon

| **Surgeon** | **SP-RATS** | **MP-RATS** | **SP-VATS** | **MP-VATS** | **Total** |
| --- | --- | --- | --- | --- | --- |
| A | 50 (16%) | 75 (24%) | 178 (58%) | 4 (1%) | 307 |
| B | 0 | 0 | 22 (61%) | 14 (39%) | 36 |
| C | 0 | 0 | 1 (50%) | 1 (50%) | 2 |

Data are presented as number of cases (row percentage).

MP, multi-port; RATS, robot-assisted thoracic surgery; SP, single-port; VATS, video-assisted thoracic surgery

Supplementary Table S2. Details of segmentectomy according to surgical method

| **Variables** | **SP RATS**  **(n = 50)** | **MP RATS**  **(n = 75)** | **VATS**  **(n = 220)** |
| --- | --- | --- | --- |
| Simple | 13 (26.0%) | 29 (38.7%) | 72 (32.7%) |
| Right-sided | 4 (8.0%) | 10 (13.3%) | 32 (14.5%) |
| RS6 | 1 (2.0%) | 9 (12.0%) | 26 (11.8%) |
| RS7+8+9+10 | 3 (6.0%) | 1 (1.3%) | 6 (2.7%) |
| Left-sided | 9 (18.0%) | 19 (25.3%) | 40 (18.2%) |
| LS1+2+3 | 1 (2.0%) | 3 (4.0%) | 10 (4.5%) |
| LS4+5 | 2 (4.0%) | 5 (6.7%) | 6 (2.7%) |
| LS6 | 5 (10.0%) | 9 (12.0%) | 21 (9.5%) |
| LS7+8+9+10 | 1 (2.0%) | 2 (2.7%) | 3 (1.4%) |
| Complex | 37 (74.0%) | 46 (61.3%) | 148 (67.3%) |
| Right-sided | 23 (46.0%) | 24 (32.0%) | 93 (42.3%) |
| RS1 | 5 (10.0%) | 5 (6.7%) | 16 (7.3%) |
| RS1+2 | 0 (0.0%) | 0 (0.0%) | 6 (2.7%) |
| RS1+3 | 0 (0.0%) | 0 (0.0%) | 2 (0.9%) |
| RS1+2+3 | 0 (0.0%) | 0 (0.0%) | 2 (0.9%) |
| RS2 | 8 (16.0%) | 8 (10.7%) | 24 (10.9%) |
| RS3 | 5 (10.0%) | 6 (8.0%) | 19 (8.6%) |
| RS3+1b | 0 (0.0%) | 0 (0.0%) | 1 (0.5%) |
| RS5 | 0 (0.0%) | 0 (0.0%) | 1 (0.5%) |
| RS6+10 | 0 (0.0%) | 0 (0.0%) | 2 (0.9%) |
| RS7 | 0 (0.0%) | 0 (0.0%) | 1 (0.5%) |
| RS8 | 2 (4.0%) | 1 (1.3%) | 5 (2.3%) |
| RS9 | 1 (2.0%) | 0 (0.0%) | 6 (2.7%) |
| RS9+10 | 0 (0.0%) | 1 (1.3%) | 0 (0.0%) |
| RS10 | 2 (4.0%) | 3 (4.0%) | 8 (3.6%) |
| Left-sided | 14 (28.0%) | 22 (29.3%) | 55 (25.0%) |
| LS1 | 2 (4.0%) | 1 (1.3%) | 4 (1.8%) |
| LS1+2 | 1 (2.0%) | 2 (2.7%) | 7 (3.2%) |
| LS1+3 | 0 (0.0%) | 0 (0.0%) | 1 (0.5%) |
| LS2 | 4 (8.0%) | 8 (10.7%) | 10 (4.5%) |
| LS3 | 4 (8.0%) | 6 (8.0%) | 19 (8.6%) |
| LS3+4 | 0 (0.0%) | 1 (1.3%) | 0 (0.0%) |
| LS3+4+5 | 0 (0.0%) | 0 (0.0%) | 1 (0.5%) |
| LS6+10 | 0 (0.0%) | 0 (0.0%) | 1 (0.5%) |
| LS7+8 | 0 (0.0%) | 1 (1.3%) | 0 (0.0%) |
| LS7+8+9 | 0 (0.0%) | 0 (0.0%) | 1 (0.5%) |
| LS8 | 2 (4.0%) | 2 (2.7%) | 3 (1.4%) |
| LS9 | 0 (0.0%) | 0 (0.0%) | 3 (1.4%) |
| LS9+10 | 0 (0.0%) | 0 (0.0%) | 1 (0.5%) |
| LS10 | 1 (2.0%) | 1 (1.3%) | 4 (1.8%) |

Data are presented as *n* (%); percentages are calculated per column.

MP, multi-port; RATS, robot-assisted thoracic surgery; SP, single-port; VATS, video-assisted thoracic surgery

Supplemental Table S3. Subgroup analysis of patients undergoing complex segmentectomy

| **Variables** | **SP-RATS** | **MP-RATS** | **VATS** | ***P*-value** | | |
| --- | --- | --- | --- | --- | --- | --- |
|  | **(n = 37)** | **(n = 46)** | **(n = 148)** | **SP vs. MP RATS** | **SP-RATS vs. VATS** | **MP-RATS vs. VATS** |
| Total operative time (min) | 135 (112–162) | 164 (129– 182) | 118 (83– 157) | 0.030 | 0.216 | < 0.001 |
| Chest tube duration (days) | 2 (1–3) | 2 (2–4) | 2 (2–4) | 0.006 | 0.045 | 1.000 |
| Postoperative hospital stays (days) | 4 (3–6) | 4 (3–6) | 4 (3–6) | 1.000 | 1.000 | 1.000 |
| Surgical margin distance (cm) | 1.7 (1.2–2.4) | 2.0 (1.4– 2.9) | 1.8 (1.2–2.3) | 0.312 | 1.000 | 0.114 |
| Complications |  |  |  | 1.000 | 1.000 | 1.000 |
| None | 33 (89%) | 38 (83%) | 129 (87%) |  |  |  |
| Minor  (Grade I–II) | 2 (5%) | 7 (15%) | 14 (9%) |  |  |  |
| Major  (≥ Grade IIIa) | 2 (5%) | 1 (2%) | 5 (3%) |  |  |  |

Data are presented as median (interquartile range) or *n* (%).

MP, multi-port; RATS, robot-assisted thoracic surgery; SP, single-port; VATS, video-assisted thoracic surgery
